# Breaking Myths: Comparable Outcomes in Lymph Node Analysis Across Surgical Methods

**DOI:** 10.3390/cancers17081312

**Published:** 2025-04-14

**Authors:** Salvatore Pezzino, Tonia Luca, Mariacarla Castorina, Giulia Fuccio Sanzà, Gaetano Magro, Stefano Puleo, Ornella Coco, Sergio Castorina

**Affiliations:** 1 Department of Medicine and Surgery, University of Enna “Kore”, 94100 Enna, Italy; salvatore.pezzino@unikore.it; 2 Mediterranean Foundation “GB Morgagni”, 95125 Catania, Italy; tluca@unict.it (T.L.); spuleo@unict.it (S.P.);; 3 Department of Medical, Surgical Sciences and Advanced Technologies “G.F. Ingrassia”, University of Catania, 95123 Catania, Italy; gmagro@unict.it

**Keywords:** colorectal cancer, lymph nodes, laparoscopic surgery, body mass index, fat clearance techniques

## Abstract

Background/Objectives: Colorectal cancer is the third most common cancer worldwide, making lymph node recovery critical for treatment decisions and prognosis. The relationship between body mass index (BMI) and the number of lymph nodes retrieved during laparoscopic and open surgeries remains controversial. This study aimed to evaluate whether surgical approach and BMI influence lymph node retrieval in colon cancer surgeries. Methods: A retrospective analysis was conducted on 560 patients who underwent colon cancer surgery at a single institution between 2018 and 2023. The average number of lymph nodes retrieved during laparoscopic and open procedures was compared. Distribution analysis using violin plots was performed to assess the pattern of lymph node yield between surgical approaches. Additionally, the impact of BMI on lymph node recovery was assessed. All surgeries were performed by a standardized surgical team using consistent fat clearance techniques. Results: The mean number of lymph nodes retrieved was 15.89 ± 0.84 for laparoscopic surgeries and 15.98 ± 0.50 for open surgeries, with no statistically significant difference (*p* = 0.9166). The violin plot analysis confirmed overlapping distributions between the two surgical approaches, with no significant difference (*p* = 0.6270). BMI also showed no significant effect on the number of lymph nodes removed during surgery. The consistency in outcomes was attributed to standardized surgical practices across all cases. Conclusions: Laparoscopic and open surgical approaches yield comparable lymph node recovery in colon cancer surgeries, both in terms of mean values and overall distribution patterns, regardless of patient BMI. These findings emphasize the importance of standardized surgical techniques in ensuring reliable outcomes and suggest that both approaches are equally effective in meeting oncological standards for lymph node retrieval.

## Simple Summary

Colorectal cancer is the third most common cancer globally, and lymph node recovery post-surgery is critical for treatment and prognosis. This retrospective study analyzed 560 colon cancer patients who underwent surgery between 2018 and 2023 to assess the impact of surgical approach and BMI on lymph node retrieval. The findings revealed no significant difference in the mean number of lymph nodes removed during laparoscopic (15.89 ± 0.84) versus open procedures (15.98 ± 0.50), with a *p*-value of 0.9166. Distribution analysis using violin plots confirmed overlapping patterns between the two surgical approaches, with no significant difference (*p* = 0.6270). Additionally, patient BMI did not significantly influence lymph node recovery. The consistency in outcomes is attributed to a standardized surgical team and fat clearance techniques, ensuring uniform results regardless of surgical method or BMI. These findings highlight the reliability of both surgical approaches in achieving comparable lymph node recovery, emphasizing the importance of standardized practices in colorectal cancer surgeries.

## 1. Introduction

Colorectal cancer (CRC) represents a significant global health issue, with etiological factors including genetics, lifestyle, obesity, and other emerging causes [1,2,3,4,5,6,7,8]. CRC ranks as the second most common cancer in Europe and is a leading cause of cancer-related deaths in the United States and Asia [9]. Accurate staging of CRC is crucial for prognosis and treatment planning, with lymph node (LN) status being a key element in this assessment [10,11]. The impact of surgical modality (laparoscopic versus open) on LN retrieval in CRC continues to be a topic of discussion. Some studies emphasize the technical difficulties associated with laparoscopic procedures, while others indicate similar LN yields between different approaches [12,13,14,15,16,17]. For instance, a meta-analysis of 15 randomized trials revealed no significant difference in LN harvest between laparoscopic and open techniques (weighted mean difference: −0.25; 95% CI: −0.57 to 0.08; *p* = 0.542), thereby confirming oncological equivalence [16]. Similarly, a multicenter study on stage II/III rectal cancer revealed comparable LN retrieval between laparoscopic and open approaches (mean lymph node count: 18.1 for laparoscopic vs. 17.9 for open; *p* = 0.721) while highlighting the greater technical complexity associated with laparoscopy in this demographic [17]. The influence of body mass index (BMI) on LN yield adds complexity to this discussion. Conflicting evidence is present, with certain studies indicating no significant differences among BMI categories, while others report an inverse relationship between BMI and LN count, implying that increased BMI may impair prognostic accuracy [17,18,19,20,21]. The observed inconsistency among studies likely indicates variability in surgical methodologies or pathological protocols, rather than biological factors [21,22,23]. Standardized fat clearance techniques have demonstrated the ability to reduce variability in LN detection, effectively addressing potential confounders such as BMI or surgical approach [24,25,26,27,28,29]. The evidence collectively indicates that LN retrieval is influenced more by procedural standardization than by patient habitus or surgical modality, thereby ensuring reproducibility across various clinical settings. Our study directly addresses this issue by examining how the surgical method (laparoscopic versus open) and BMI affect LN retrieval, with a particular emphasis on the relevance of standardized fat clearance protocols. We analyzed 560 patients who underwent colorectal cancer surgery from 2018 to 2023 to demonstrate that standardized surgical techniques and fat clearance procedures, rather than surgical approach or patient BMI, are the key factors influencing adequate LN harvesting.

## 2. Materials and Methods

### 2.1. Study Design and Setting

This study sought to evaluate the influence of body mass index (BMI) on the number of lymph nodes harvested after colorectal cancer surgery, with a particular focus on comparing laparoscopic and open surgical techniques. The study was a retrospective analysis performed at a single institution. The surgical team exhibited a notable degree of consistency, with one surgeon overseeing both open and laparoscopic procedures, and a single pathologist tasked with doing histopathological examinations. All procedures adhered to complete mesocolic excision (CME) principles for colon cancer and total mesorectal excision (TME) principles for rectal cancer, ensuring en-bloc resection of the tumor with its lymphatic basin. Lymph node retrieval was performed by a dedicated gastrointestinal pathologist using a standardized fat clearance protocol. The retrieval of lymph nodes was performed as previously reported [30]. Briefly, the entire colorectal specimens were fixed for 48 h in a modified Koren solution with a mixture of 40% ethanol, 40% ether, 10% acetic acid, and 10% formaldehyde. Reagent were purchased from Sigma-Aldrich Co. (Milan, Italy). After fat clearance, the lymph nodes were easily identified as white foci against yellow and translucent adipose tissue.

### 2.2. Patient Selection

We considered patients who received colorectal cancer surgery at our institution from 1 January 2018 to 31 December 2023. Patients were discovered through the hospital’s electronic medical records and cancer registry. The inclusion criteria encompassed persons aged 18 years or older diagnosed with colorectal adenocarcinoma who received either laparoscopic or open surgery with a curative aim. The exclusion criteria eliminated individuals presenting with metastatic disease, those undergoing emergency procedures, and those with insufficient medical data. Due to the retrospective nature of the study, the informed consent of the patients was not required because the study analyzed the anonymous clinical data of the patients. The selection of the surgical approach (laparoscopic vs. open) was determined by the following standardized factors applied uniformly throughout the study period: the extension of neoplasm, cancer staging, anesthesiology consultation, patient age, and cardiopulmonary status. Despite the retrospective nature of the analysis, the risk of chronological bias was minimized by the constant application of standardized surgical and pathological protocols. The decision-making process for the choice of surgical approach remained unchanged during the entire study period. The analysis focused on comparing the outcomes of laparoscopic and open approaches rather than evaluating temporal trends, further limiting the impact of the chronological sequence on the validity of the results. Therefore, the retrospective design does not significantly compromise the conclusions of the study.

### 2.3. Data Collection

Data were retrospectively gathered from patient medical records, surgical reports, and pathology reports. The gathered data encompassed patient demographics (age, sex), the BMI at the time of surgery, the type of surgical operation (laparoscopic or open), and the total count of lymph nodes removed.

### 2.4. Outcome Measures

The principal outcome measure was the quantity of lymph nodes excised during the surgical procedure. The secondary outcomes encompassed the sufficiency of lymph node retrieval (defined as 12 or more lymph nodes) [31,32], the impact of BMI on lymph node yield, and the comparison of laparoscopic versus open surgical techniques for lymph node harvest.

### 2.5. Statistical Analysis

The descriptive data were presented as the mean, with the standard error of the mean (SEM) when relevant. An unpaired Student’s *t*-test was conducted to compare the mean number of nodes extracted from laparoscopic and open operations. To visualize and compare the lymph node yield distribution between the two surgical approaches, violin plots were generated; the Mann–Whitney U test was used to compare these distributions, ensuring robustness to potential non-normality. Linear regression analysis was performed to assess the relationship between BMI and the quantity of collected lymph nodes. To evaluate the influence of BMI on lymph node yield, we conducted a subgroup analysis, stratified by surgical method (laparoscopic versus open). A *p*-value < 0.05 was considered statistically significant for all analyses. All statistical analyses and graphical representations were performed using GraphPad Prism software (version 10.0.2, GraphPad Software, San Diego, CA, USA).

## 3. Results

All patients’ clinical characteristics are displayed in Table 1. The cohort consisted of 560 patients who underwent colorectal cancer surgery between 2018 and 2023. Among these, 166 patients (29.6%) underwent laparoscopic surgery, while 394 patients (70.4%) underwent open surgery. Regarding sex distribution, the laparoscopic group included 81 males (48.8%) and 85 females (51.2%), whereas the open group comprised 177 males (44.9%) and 217 females (55.1%). The mean age of the patients in the laparoscopic group was 64.46 ± 0.87 years, compared to the open surgery group, where the mean age was 74.28 ± 0.21 years. The mean body mass index (BMI) was slightly higher in the laparoscopic group (26.01 ± 0.29 kg/m^2^) compared to the open group (25.48 ± 0.47 kg/m^2^). In terms of surgical procedures, right hemicolectomy was predominantly performed in the open group, with 212 cases (53.8%) compared to only 27 cases (16.3%) in the laparoscopic group. Left hemicolectomy was more evenly distributed, with 88 cases (53%) in the laparoscopic group and 121 cases (30.7%) in the open group. Anterior resection of the rectum accounted for 51 cases (30.7%) in the laparoscopic group and 61 cases (15.5%) in the open group.

**Figure 1:**
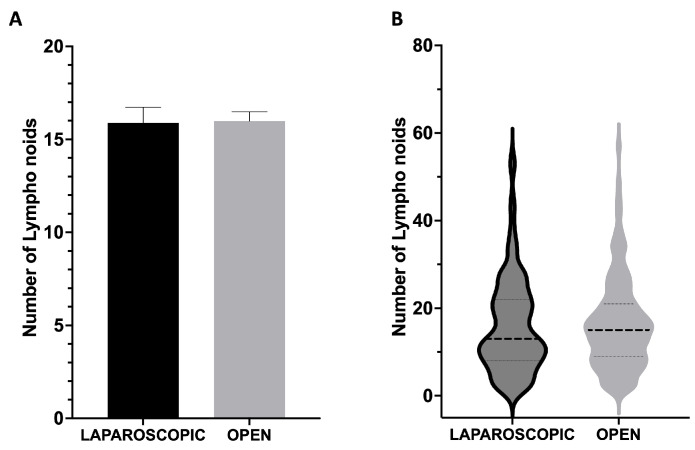
Lymph node retrieval in laparoscopic versus open colorectal cancer surgery. (**A**) The mean number of lymph nodes collected during laparoscopic and open surgeries: 15.89 ± 0.84 and 15.98 ± 0.50, respectively (*p* = 0.9166). (**B**) Violin plots displaying the distribution of lymph node yield for both surgical approaches. The horizontal dashed lines within each violin represent the median (center line, laparoscopic: 13.00, open: 15.00) and the 25th (laparoscopic: 8.00, open: 9.00) and 75th percentiles (laparoscopic: 22.00, open: 21.00); range values: 1–54 (laparoscopic) and 1–57 (open) lymph nodes; (*p* = 0.6270).

The study findings, illustrated in Figure 1, demonstrate that there is no significant difference in the number of lymph nodes harvested when comparing laparoscopic and open surgical methods. Data analysis reveals that the mean number of lymph nodes retrieved was 15.89 ± 0.8413 for laparoscopic procedures and 15.98 ± 0.5022 for open procedures, with a *p*-value of 0.9166, indicating that the selected surgical approach does not significantly influence lymph node yield (Figure 1A). To further investigate this observation, an analysis of lymph node distribution was conducted using violin plots (Figure 1B). These plots highlight the overlapping distributions between the two surgical approaches, visually confirming the comparability of the results. An analysis of the median values showed 13.00 lymph nodes for the laparoscopic approach and 15.00 for the open approach. The violin plots further reveal that the 25th percentile was 8.00 for laparoscopy and 9.00 for open surgery, while the 75th percentile was 22.00 for laparoscopy and 21.00 for open surgery. The overall range varied from 1 to 54 lymph nodes in laparoscopic surgery and from 1 to 57 in open surgery. The Mann–Whitney test confirmed the absence of statistically significant differences between the two distributions (*p* = 0.6270). This consistency in results, in terms of both mean values and overall distribution, suggests that both surgical approaches are equally effective in ensuring adequate lymph node retrieval, a crucial aspect of proper staging and treatment of colorectal cancer.

**Figure 2:**
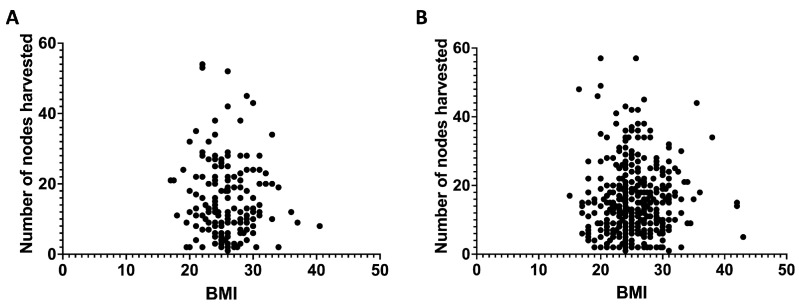
The correlation between body mass index and lymph nodes harvested (period from 2018–2023) among all patients undergoing laparoscopic surgery (**A**) or open surgery (**B**). (**A**): r = −0.09143 and *p* = 0.2414; (**B**): r = 0.0009955 and *p* = 0.9843.

Figure 2 depicts the relationship between a patient’s BMI and the number of lymph nodes collected during laparoscopic surgery (Figure 2A) and open surgery (Figure 2B). The scatterplots illustrate individual data points, displaying BMI values (kg/m^2^) on the *x*-axis and the quantity of lymph nodes retrieved on the *y*-axis. Each dot signifies an individual patient, facilitating the visualization of distribution and variability within the cohort. The analysis included medical records from 560 patients who underwent colorectal cancer surgery from 2018 to 2023. Figure 2A includes patients who received laparoscopic surgery (166 cases), whereas Figure 2B encompasses those who underwent open surgery (394 cases). The scatterplots indicate no discernible trend or correlation between BMI and lymph node yield, regardless of the surgical modality employed. Linear regression analysis indicated no significant relationship between BMI and lymph node retrieval in both groups (laparoscopic: r = −0.09143, *p* = 0.2414; open: r = 0.0009955, *p* = 0.9843). The results indicate that BMI does not affect lymph node yield, irrespective of the surgical method employed.

**Figure 3:**
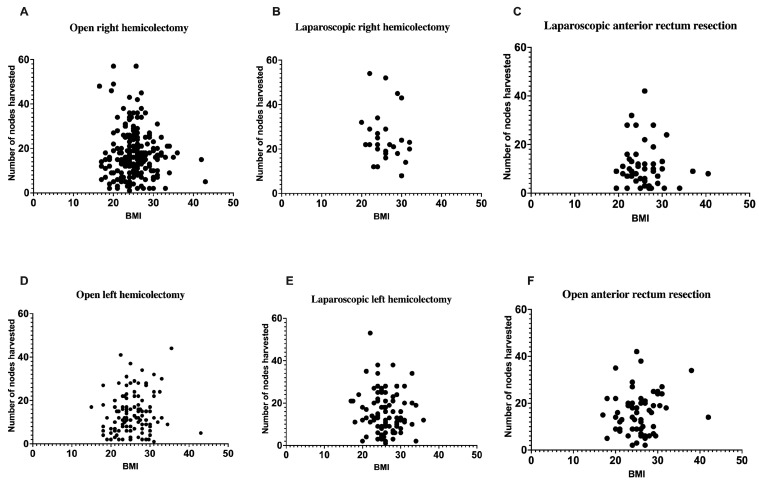
The correlation between a patient’s body mass index and the number of lymph nodes harvested (period from 2018–2023) during laparoscopic or open surgery, namely right hemicolectomy, left hemicolectomy, and anterior rectum resection. (**A**): r = −0.07447 and *p* = 0.2804; (**B**): r = −0.1501 and *p* = 0.4548; (**C**): r = −0.03450 and *p* = 0.8101; (**D**): r = 0.1065 and *p* = 0.2448; (**E**): r = −0.1246 and *p* = 0.2473; (**F**): r = 0.1345 and *p* = 0.3016.

Figure 3 represents the correlation between a patient’s BMI and the number of lymph nodes retrieved during various colorectal surgical procedures, encompassing both laparoscopic and open techniques for right hemicolectomy (Figure 3A,B), anterior rectum resection (Figure 3C,F), and left hemicolectomy (Figure 3D,E). The scatterplots depict individual data points, with BMI values (kg/m^2^) plotted on the *x*-axis and the quantity of lymph nodes harvested on the *y*-axis. The panels are categorized by surgical approach, facilitating direct comparisons within each procedure type. Figure 3A,D,F depict open surgeries, whereas Figure 3B,C,E illustrate laparoscopic surgeries. No significant correlation exists between BMI and lymph node yield across all surgical methods and procedure types. Statistical analysis via linear regression indicated no significant relationships between BMI and lymph node retrieval across all subgroups, with all *p*-values surpassing 0.05. The findings indicate that neither BMI nor surgical approach has a significant impact on the number of lymph nodes retrieved during colorectal cancer surgeries. This result is consistent with the conclusions derived from Figure 2, which examines the overall relationship between BMI and lymph node yield without differentiating by specific procedure type.

## 4. Discussion

Effective therapy of colorectal cancer (CRC) necessitates precise staging of the illness to ascertain suitable treatment options. Lymph node (LN) involvement is a pivotal aspect of CRC staging, and the increased lymph node yield correlates with more precise tumor staging even if the association between lymph node yield and survival, especially in node-negative patients, is complex and not fully clarified [29,30,31,32,33,34,35,36,37].

The impact of surgical technique (laparoscopic versus open) on LN retrieval in colorectal cancer remains contentious [12,13,14,15,16,17]. The outcomes of our study demonstrate a consistent pattern of LN retrieval in both laparoscopic and open colorectal cancer operations. As illustrated in Figure 1, the mean values (15.89 ± 0.84 for laparoscopic surgeries and 15.98 ± 0.50 for open surgeries) show remarkable similarity (*p* = 0.9166), suggesting that the surgical method does not substantially affect lymph node yield. This finding is further strengthened by our violin plot analysis (Figure 1B), which provides a more comprehensive visualization of the complete distribution of lymph node yields beyond simple mean comparisons. This analysis confirms the comparability between the two approaches, showing overlapping distributions with median values of 13.00 for laparoscopic and 15.00 for open procedures. The Mann–Whitney test revealed no significant difference between these distributions (*p* = 0.6270), further supporting the oncological equivalence of both approaches. The similar interquartile ranges (25th percentile: 8.00 for laparoscopic, 9.00 for open; 75th percentile: 22.00 for laparoscopic, 21.00 for open) provide additional evidence of comparable lymph node harvest patterns regardless of surgical technique. These findings align with previous research demonstrating that laparoscopic surgery provides adequate lymph node dissection comparable to open surgery [12,13,14,15,16,17,38,39,40,41,42,43].

As aforementioned, conflicting evidence exists, with certain studies indicating no significant variations among BMI categories, while others reveal an inverse association between BMI and LN count, implying that an increased BMI may impair prognostic accuracy [12,13,14,15,16,17]. Our data indicate that BMI does not influence the quantity of LN extracted during surgery. As shown in Figure 2, the correlation analysis between BMI and lymph node yield revealed no significant relationship in either laparoscopic (r = −0.09143, *p* = 0.2414) or open surgeries (r = 0.0009955, *p* = 0.9843). This finding is further supported by the detailed subgroup analyses in Figure 3, where various surgical procedures (right hemicolectomy, left hemicolectomy, and anterior rectum resection) similarly showed no significant correlation between BMI and lymph node yield regardless of the surgical approach. Specifically, the analysis revealed no significant correlation across all procedure types: laparoscopic right hemicolectomy (r = −0.1501, p = 0.4548), open right hemicolectomy (r = −0.07447, *p* = 0.2804), laparoscopic left hemicolectomy (r = −0.1246, *p* = 0.2473), open left hemicolectomy (r = 0.1065, *p* = 0.2448), laparoscopic anterior rectum resection (r = −0.03450, *p* = 0.8101), and open anterior rectum resection (r = 0.1345, *p* = 0.3016). This comprehensive subgroup analysis provides robust evidence that patient BMI does not affect lymph node retrieval regardless of anatomical location and surgical technique. This finding aligns with recent research indicating that lymph node retrieval is unaffected by BMI [16,43,44,45,46]. Given the increasing prevalence of overweight/obesity worldwide and its established role as a risk factor for colorectal cancer, our results suggest that patient BMI should not be a limiting factor in the surgical management of colorectal cancer, particularly in terms of lymph node harvest.

A crucial factor of our success in LN retrieval is the application of standardized fat clearance protocols by our medical staff. The consistency in LN yield across both laparoscopic and open surgeries, irrespective of patient BMI or tumor location, underscores the dominance of methodological standardization over surgical technique or patient-related variables. This conclusion is reinforced by multiple studies demonstrating the efficacy of fat clearance techniques in minimizing variability and enhancing LN detection.

For instance, Yeh et al. (2021) demonstrated that an improved fat clearing protocol increased LN retrieval from 14.7 ± 6.2 to 20.8 ± 9.0 nodes per specimen (*p* < 0.05), directly improving compliance with staging guidelines (≥12 LNs) from 80% to 91% of cases [28]. Similarly, Wang et al. (2009) reported a dramatic increase in LN yield (from 5.2 ± 0.6 to 20.4 ± 1.2 nodes, *p* < 0.001) in rectal cancer patients post-neoadjuvant therapy, highlighting the technique’s utility in overcoming challenges posed by treatment-induced tissue changes [47].

The clinical impact of these protocols is further validated by Kajitani et al. (2024), who demonstrated that enzymatic fat dissolution significantly improved the detection of small lymph nodes (<5 mm) during colon cancer surgery, which are often missed with manual palpation methods. This enhanced detection allowed for more accurate staging and potentially influenced treatment decisions, reinforcing the role of standardized fat clearance techniques in modern colorectal cancer management [48]. Similarly, Maeda et al. (2018) highlighted the importance of modern fat-dissociation methods for improving LN retrieval in obese patients, where dense adipose tissue can obscure smaller nodes during conventional pathological examination [26].

Our findings, coupled with the existing literature, contest the conventional focus on surgical approach or BMI as primary determinants of LN yield. For example, in our cohort, laparoscopic and open right hemicolectomies showed comparable LN distributions (median: 13 vs. 15 nodes, *p* = 0.627), while BMI exhibited no correlation with yield (laparoscopic: r = −0.091, *p* = 0.24; open: r = 0.001, *p* = 0.98).

On the other hand, the consistency in LN yield, irrespective of the surgical method or patient BMI, can be ascribed to the uniformity of our medical staff. This uniformity reflects the team’s overall knowledge and the standardized protocols developed, guaranteeing that each patient receives optimal care customized to their unique needs while upholding uniform surgical and pathological standards. The team consisted of one surgeon and one pathologist, tasked with performing both open and laparoscopic procedures and conducting histological studies, respectively. This conclusion is corroborated by the current literature highlighting the significance of collaboration in surgical environments for enhancing patient outcomes [49,50,51].

Nonetheless, it is crucial to recognize the constraints of our research. The patient cohort exhibited heterogeneity regarding obesity, with a mean BMI of roughly 26, suggesting that most patients were classified as overweight rather than obese. The absence of homogeneity in patient BMI may affect the generalizability of our findings to groups with elevated obesity levels. Moreover, as a retrospective study, there may be inherent biases due to the non-randomized selection of patients and the potential for missing or incomplete data. Additionally, the findings from a single center may not be generalizable to other settings.

## 5. Conclusions

Laparoscopic and open surgical approaches demonstrate similar lymph node retrieval in colon cancer surgeries, with comparable mean values and distribution patterns, independent of patient body mass index (BMI). The findings underscore the significance of standardized surgical techniques in achieving reliable outcomes and indicate that both approaches are comparably effective in achieving the oncological standards for lymph node retrieval. Conversely, the constant lymph node (LN) yield regardless of surgical approach or patient body mass index is likely due to the consistency of our medical personnel. While our study did not directly compare different specimen processing techniques, the observed consistency in LN yield across surgical approaches and BMI categories strongly suggests that standardized fat clearance protocols may significantly contribute to ensuring reliable oncological staging, independent of surgical modality or patient characteristics. This hypothesis is supported by the existing literature on fat clearance efficacy and warrants further investigation through direct comparative studies.

## Figures and Tables

**Table 1:** Clinical characteristics of 560 patients (2018–2023 years); n. pts = number of patients; mean ± SEM.

	Laparoscopic	Open
Number of patients	166	394
Sex (male) (n. pts)	81	177
Sex (female) (n. pts)	85	217
Age (mean ± SEM)	64.46 ± 0.87	74.28 ± 0.21
BMI (kg/m^2^) (mean ± SEM)	26.01 ± 0.29	25.48 ± 0.47
Right hemicolectomy (n. pts)	27	212
Left hemicolectomy (n. pts)	88	121
Anterior resection of rectum (n. pts)	51	61

## Data Availability

The authors confirm that the data supporting the findings of this study are available within the article.
